# Understanding the Central Role of Citrate in the Metabolism of Cancer Cells and Tumors: An Update

**DOI:** 10.3390/ijms22126587

**Published:** 2021-06-19

**Authors:** Philippe Icard, Antoine Coquerel, Zherui Wu, Joseph Gligorov, David Fuks, Ludovic Fournel, Hubert Lincet, Luca Simula

**Affiliations:** 1 Medical School, Université Caen Normandie, CHU de Caen, 14000 Caen, France; antoine.coquerel@gmail.com; 2 UNICAEN, INSERM U1086 Interdisciplinary Research Unit for Cancer Prevention and Treatment, Normandie Université, 14000 Caen, France; 3 Service de Chirurgie Thoracique, Hôpital Cochin, Hôpitaux Universitaires Paris Centre, APHP, Paris-Descartes University, 75014 Paris, France; ludovic.fournel@aphp.fr; 4 INSERM U1075, COMETE Mobilités: Attention, Orientation, Chronobiologie, Université Caen, 14000 Caen, France; 5 School of Medicine, Shenzhen University, Shenzhen 518000, China; wuzherui@gmail.com; 6 Oncology Department, Tenon Hospital, Pierre et Marie Curie University, 75020 Paris, France; joseph.gligorov@aphp.fr; 7 Service de Chirurgie Digestive et Hépato-Biliaire, Hôpital Cochin, Hôpitaux Universitaires Paris Centre, APHP, Paris-Descartes University, 75014 Paris, France; david.fuks@aphp.fr; 8 Descartes Faculty of Medicine, University of Paris, Paris Center, 75006 Paris, France; 9 INSERM U1052, CNRS UMR5286, Cancer Research Center of Lyon (CRCL), 69008 Lyon, France; hubert.lincet@univ-lyon1.fr; 10 ISPB, Faculté de Pharmacie, Université Lyon 1, 69373 Lyon, France; 11 Department of Infection, Immunity and Inflammation, Institut Cochin, INSERM U1016, CNRS UMR8104, University of Paris, 75014 Paris, France; luca.simula@inserm.fr

**Keywords:** citrate, cancer cells, Warburg effect, ACLY, resistance to therapies

## Abstract

Citrate plays a central role in cancer cells’ metabolism and regulation. Derived from mitochondrial synthesis and/or carboxylation of α-ketoglutarate, it is cleaved by ATP-citrate lyase into acetyl-CoA and oxaloacetate. The rapid turnover of these molecules in proliferative cancer cells maintains a low-level of citrate, precluding its retro-inhibition on glycolytic enzymes. In cancer cells relying on glycolysis, this regulation helps sustain the Warburg effect. In those relying on an oxidative metabolism, fatty acid β-oxidation sustains a high production of citrate, which is still rapidly converted into acetyl-CoA and oxaloacetate, this latter molecule sustaining nucleotide synthesis and gluconeogenesis. Therefore, citrate levels are rarely high in cancer cells. Resistance of cancer cells to targeted therapies, such as tyrosine kinase inhibitors (TKIs), is frequently sustained by aerobic glycolysis and its key oncogenic drivers, such as Ras and its downstream effectors MAPK/ERK and PI3K/Akt. Remarkably, in preclinical cancer models, the administration of high doses of citrate showed various anti-cancer effects, such as the inhibition of glycolysis, the promotion of cytotoxic drugs sensibility and apoptosis, the neutralization of extracellular acidity, and the inhibition of tumors growth and of key signalling pathways (in particular, the IGF-1R/AKT pathway). Therefore, these preclinical results support the testing of the *citrate strategy* in clinical trials to counteract key oncogenic drivers sustaining cancer development and resistance to anti-cancer therapies.

## 1. Introduction

Citrate is produced from oxaloacetate (OAA) and acetyl-CoA by citrate synthase (CS) in mitochondria, and it plays a central role in both normal and cancer cell metabolism [1]. In mitochondria, as an intermediate of the tricarboxylic acid (TCA) cycle (also known as the Krebs cycle), citrate sustains the generation of reduced nicotinamide adenine dinucleotide (NADH) and flavine adenine dinucleotide (FADH_2_) molecules, which lead to ATP production via ATP synthase, the complex V of the Electron Transport Chain (ETC). Remarkably, citrate plays several additional roles in cells [1]. Indeed, it can be exported into the cytosol, where it is converted by ATP-citrate lyase (ACLY) back into acetyl-CoA and OAA. Cytosolic acetyl-CoA sustains fatty acid (FA) synthesis (FAS), and it also regulates the activities of proteins, particularly histones (required for DNA transcription), through acetylation. Cytosolic OAA can be converted into phosphoenolpyruvate (PEP) to generate glucose via gluconeogenesis, or it can sustain nucleotide and polyamine synthesis after transformation into aspartate. Therefore, citrate is a gauge of nutrients available for biosynthesis and ATP production generated via oxidative phosphorylation (OXPHOS). In addition, citrate is a key regulatory molecule, which targets (directly or indirectly) catabolic and anabolic pathways (fatty acid β-oxidation (FAO) and FAS, glycolysis, and gluconeogenesis) in a manner such that when one pathway is activated, the other is inhibited. For example, citrate directly inhibits the main regulators of glycolysis, phosphofructokinase-1 (PFK1) and phosphofructokinase-2 (PFK2) [2,3], while it enhances gluconeogenesis by promoting fructose-1,6-biphosphatase (FBPase) [4].

An example of the key role of citrate in normal cells can be found in hepatocytes. In these non-proliferative cells, when glucose is abundant (such as after meal), insulin promotes the conversion of glucose into acetyl-CoA, and then citrate [5,6]. Subsequently, citrate is exported in the cytosol, sustaining FAS in hepatocytes and leading to fat storage in adipocytes via lipoproteins [5]. In contrast, when the glucose serum level is low, the hormone glucagon promotes lipolysis and subsequent FAO in liver. FAO transforms FAs into acetyl-CoA, which is then converted by CS into citrate, fuelling the TCA cycle coupled to ATP production. In addition, glucagon promotes gluconeogenesis, a pathway consuming ATP, to supply glucose for the brain and erythrocytes [5,7]. Different sources can be used to obtain the carbons required to generate glucose by gluconeogenesis, such as lactate, amino acids (in particular, alanine), acetone (a ketone body (KB)), or glycerol (derived from the hydrolysis of triglycerides). Importantly, in the context of FAO-coupled gluconeogenesis, the high cytosolic concentration of citrate (resulting from its mitochondrial export) directly inhibits the glycolytic enzymes PFK2 and PFK1 [8]. Thus, through this so-called Randle cycle regulation [7], glycolysis is nearly switched off, while fat degradation, gluconeogenesis and the production of glucose are favoured [2]. Moreover, citrate is involved in numerous physiological processes and regulations, such as insulin secretion [9], prostatic fluid and spermatozoid mobility [10], the formation and strength of dental and bone tissues [11,12], immune and inflammatory response, and anti-bacterial defence [13,14,15,16,17]. Consequently, the deregulation of citrate metabolism is observed in various pathologies, particularly obesity, diabetes, inflammation, and cancer. Interestingly, citrate is used for disinfection and in food preservatives against Clostridium botulinum, an anaerobic Gram-positive pathogen [18]. In addition, citrate shows antibacterial activity against other proliferative gram-positive species, such as Staphylococci, and yeast-like fungi, such as Candida albicans [19,20].

In proliferative cancer cells, which are not censored by suppressive controls, a metabolic deregulation is promoted by the high metabolic turnover, resulting in the maintenance of citrate in a low concentration range, a mechanism that we have considered to be a key driving force for the Warburg effect (i.e., enhanced aerobic glycolysis with lactic acid production even in the presence of oxygen) [1,21,22,23]. Several years ago, we were the first to demonstrate that citrate, a well-known inhibitor of PFK and the Pasteur effect (i.e., anaerobic fermentation in yeast) [24], inhibits the proliferation of various cancer cells of solid tumors (human mesothelioma, gastric and ovarian cancer cells) at high concentrations (10–20 mM), promoting apoptosis and the sensitization of cells to cisplatin [25,26,27].

In line with our hypothesis that a low citrate level promotes the Warburg effect, it has been shown that the inhibition of paclitaxel-resistant lung cancer cells’ proliferation by dichloroacetate is associated with increasing cytosolic citrate concentration [28]. Moreover, citrate level was found depleted in malignant mesothelioma cells (compared to non-malignant cells) [29], and microRNA-126, which suppresses tumor growth, also restored citrate level through the inhibition of ACLY and the Protein Kinase B (also named Akt), a pathway promoting ACLY activation [29].

In this review, we will synthetize the preclinical results of the literature on citrate in cancer cells and we will try to explain or answer several questions. Why does a low concentration of citrate promote the Warburg effect and cancer aggressiveness? Which level of citrate can be expected in cells preferentially relying on oxidative metabolism [30,31]? What could be the impact on the tumor microenvironment (TME) of citrate administration at high doses? Lastly, which *citrate strategy* do we recommend for testing in clinical trials?

## 2. The Central Role of Citrate in the Warburg Effect

In normal cells, such as liver cells, citrate regulates several strategic enzymes of both catabolic and anabolic pathways. Indeed, citrate inhibits the main glycolytic enzymes PFK1 and PFK2, while at the same time it enhances the activity of the enzymes acetyl-CoA carboxylase (ACC) (driving FAS) and fructose-1,6-bisphosphatase (FBPase) (promoting gluconeogenesis) [2,8]. Thus, citrate acts as a gauge, which switches off glycolysis and concomitantly switches on gluconeogenesis and FAS.

Of note, malonyl-CoA (a derivate of citrate) further sustains FAS and inhibits its inverse metabolic pathway (i.e., the FAO) through a direct downregulation of the carnitine acyltransferase 1 (CPT1), the enzyme responsible for the transfer of FA-derived acyl-CoA into mitochondria [32]. As we will see, these regulations are impaired in cancer cells, and a low citrate level, resulting from the rapid turnover of molecules, appears as a key driver of the Warburg effect.

### 2.1. The Warburg Effect

Numerous cancer cells rely on aerobic glycolysis, a metabolism first identified by Otto Warburg [23], and extensively studied in recent years [33,34], in particular to understand how it can promote cancer cells’ aggressiveness and drug resistance [21,35]. Briefly, elevated glucose uptake sustains biosynthesis since glycolysis and its branched pathways support the synthesis of precursors of several macromolecules. By a cascade of ten enzymatic reactions, glucose is transformed into pyruvate, and a great part of this pyruvate is converted by lactate dehydrogenase A (LDHA) into lactate. However, in cancer cells, the re-expression of pyruvate kinase (PK) in its embryonic dimeric form PKM2, which is less active than the tetrameric form, creates a “bottleneck” at the end of glycolysis, promoting the accumulation of upstream glycolytic intermediates. Consequently, these intermediates can feed branched pathways, in particular the glycerol pathway, required for lipid synthesis, and the glycine–serine–methionine pathway, required for nucleotide and polyamine synthesis. Glucose metabolism can be also channelled towards the pentose phosphate pathway (PPP), whose oxidative part generates the reduced nicotinamide adenine dinucleotide phosphate (NADPH, H^+^) required for redox balance, nucleotide synthesis and FAS. Of note, lysine acetylation of metabolic enzymes is a key posttranslational regulation, which adapts the activities of the metabolic enzymes to nutrients availability, as showed in human liver tissue [36]. In cancer cells, a high glucose consumption promotes PKM2 acetylation on lysine 305, and thus its inactivation [37]. Monomeric PKM2 (less active than tetrameric PKM2) also translocates into the nucleus during G1 phase, promoting concomitant activation of Myelocytomatosis Viral Oncogene (*MYC*) and cell cycle progression, resulting in the stimulation of glycolytic gene expression and active transcription of hypoxia-inducible factor-1α (HIF-1α), a key inducer of the Warburg effect (for references, see [38]). It is worth noting that cancer cells metabolize glutamine too, which, once transformed into glutamate and then α-ketoglutarate (AKG), sustains the TCA cycle and the mitochondrial production of ATP. However, the complete oxidation of one molecule of glutamine results in less ATP molecules than the complete oxidation of glucose and palmitate (9 ATP, 36 ATP and 106 ATP, respectively). Importantly, glutamine metabolism—coupled with glycolysis—provides nitrogen groups and aspartate, which sustains nucleotide and polyamines synthesis.

**Figure 1:**
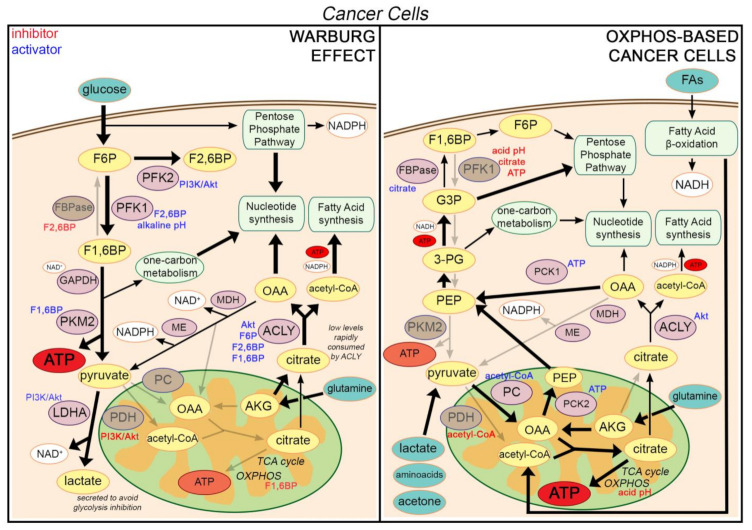
**Citrate fate in cancer cells.** Enzymes are indicated in pink (active) or brown (inactive). Metabolites are in yellow (intracellular) or green (when uptaken from the extracellular environment). Key cellular processes are in light green. Specific regulators of protein activities are indicated in blue (activators) or red (inhibitors). Arrow size is proportional to pathway engagement (in gray if the pathway is not active). Differences in glycolytic and mitochondrial ATP production are highlighted using a different font size. Small ATP and NAD(P)H boxes (not originating from an arrow) indicate their requirement to sustain the corresponding cellular processes (FAS from acetyl-CoA, 3-PG to G3P conversion, and GAPDH activity). On the left side, the functioning of cells relying on the Warburg effect is summarized. Glucose is channelled into glycolysis to produce ATP. Moreover, a portion of glycolytic intermediates is diverted toward the PPP and the serine one-carbon metabolism to sustain nucleotide synthesis. Since pyruvate is preferentially transformed by LDHA into lactate (with regeneration of NAD^+^ required for GAPDH functioning), it poorly supports the generation of citrate from acetyl-CoA and OAA in mitochondria. Consequently, citrate can be derived mainly from glutamine via AKG. Moreover, citrate is rapidly converted by ACLY into OAA (sustaining nucleotide synthesis and/or being recycled into malate and pyruvate) and acetyl-CoA (sustaining lipid synthesis and histone acetylation). On the right side, the functioning of cells relying on oxidative metabolism is depicted, in particular FAO, a condition observed especially in case of glucose deprivation. High levels of mitochondrial acetyl-CoA (derived from FAO) and OAA (derived from various carbon sources being converted into pyruvate) sustains the production of high levels of citrate. Citrate is used to a minor extent in the TCA cycle to support ATP synthesis, and it is mainly exported in the cytosol to sustain the synthesis of nucleotides and de novo FAS, as seen in glucose-based cancer cells. However, cytosolic OAA can be also diverted towards the production of PEP to feed gluconeogenesis. The latter pathway is required to support nucleotide synthesis via PPP and serine one-carbon metabolism. Of note, oscillatory levels of citrate may promote futile cycles, such as the alternance between glycolysis (when citrate is low) and gluconeogenesis (when citrate is high) or between FAO (promoted by low citrate via low malonate levels) and FAS (promoted by high citrate via high malonate levels). Furthermore, high consumption of citrate to sustain biosynthesis can induce a low citrate level, promoting a truncated gluconeogenesis due to FBPase inactivation (not figured). ***Abbreviations:*** ACLY: ATP citrate lyase, AKG: α-ketoglutarate (also known as 2-oxoglutarate), AKT: Protein Kinase B, F1,6P: Fructose-1,6-bisphosphate, F2,6BP: Fructose-2,6-biphosphate, F6P: Fructose 6-phosphate, FAs: Fatty acids, FAO: Fatty acid β-oxidation, FAS: Fatty acid synthesis, FBPase: fructose-1,6-bisphosphatase, G3P: Glyceraldehyde-3-phosphate, LDHA: Lactate dehydrogenase A, MDH: Malate dehydrogenase, ME: Malic enzyme, NAD^+^: Nicotinamide adenine dinucleotide oxidized, NADPH: Nicotinamide adenine dinucleotide phosphate, OAA: Oxaloacetate, OXPHOS: Oxidative phosphorylation, PC: Pyruvate carboxylase, PDH: Pyruvate dehydrogenase, PEP: Phosphoenolpyruvate, PCK: Phosphoenolpyruvate carboxylase, PFK1: Phosphofructokinase 1, 3-PG: 3 Phosphoglycerate, PI3K: phosphatidylinositol-3-kinase, PPP: pentose phosphate pathway, PKM2: Pyruvate kinase muscle embryonic isozyme 2, TCA cycle: tricarboxylic acid cycle.

In these aerobic glycolysis-based cancer cells, citrate can be generated in mitochondria from acetyl-CoA and OAA by CS. While a reduced flux of pyruvate into mitochondria is still able to provide the required acetyl-CoA (a major part of pyruvate being converted into lactate), OAA is presumably mainly derived from the glutamine through the conversion into AKG (Figure 1). However, in hypoxic cancer cells or in cells harbouring mutations of enzymes in the TCA cycle or in the ETC, an alternative reductive glutamine-dependent pathway can be the dominant means of citrate production. In this case, the carboxylation of AKG derived from glutamate leads directly to isocitrate, and then citrate, without the involvement of acetyl-CoA and OAA [39]. 

In highly glycolytic cells, the mitochondrial production of ATP is reduced. This is due to the inhibition of the OXPHOS by the high levels of the glycolytic intermediate fructose-1,6-bisphosphate (F-1,6-BP), a process likely explaining the Crabtree effect, i.e., the inhibition of cell respiration by high concentrations of glucose [40]. Therefore, the carbon flux in the TCA cycle is downregulated, and citrate is mainly exported in the cytosol, where it is transformed by ACLY into acetyl-CoA and OAA. Importantly, mitochondrial aconitase (ACO2) (the enzyme metabolizing citrate in the TCA cycle) maintains a high citrate/isocitrate ratio, thus favoring the export of citrate into the cytosol, particularly in prostate epithelial cells [41]. Therefore, ACO2 plays a key regulatory role in mitochondria, either sustaining the functioning of the TCA cycle (and thus, ATP production) or promoting the efflux of citrate (coupled with histone acetylation and/or FAS) [42,43,44,45]. Of note, cytosolic OAA can be converted into malate by malate-dehydrogenase (MDH), this reaction regenerating NAD^+^ from NADH, a cofactor required for the functioning of glycolysis at the glyceraldehyde-3-phosphate dehydrogenase (GAPDH) level. Then, malate derived from OAA enters into mitochondria in exchange with citrate via the mitochondrial citrate/isocitrate carrier (CIC), also known as SLC25A1 [46]. Alternatively, malate can be converted by malic enzyme (ME) into pyruvate, this reaction producing NADPH, H^+^. OAA can be also transformed by cytosolic phosphoenolpyruvate carboxykinase (PCK1) into PEP (which sustains gluconeogenesis), or by aspartate aminotransferase (AST) into aspartate (which sustains nucleotide synthesis and polyamine synthesis) (Figure 1).

In the TME, hypoxia may further promote the Warburg effect in cancer cells. Indeed, the main hypoxia-induced transcription factor, HIF-1α, upregulates glucose membranes transporters (GLUT1, GLUT3) and several key glycolytic enzymes, such as hexokinase 2 (HK2), PFK, phosphoglycerate kinase1 (PGK1), PKM2, PFK2 and LDHA [47]. In addition, HIF-1α activates the gene encoding the kinase pyruvate dehydrogenase kinase 1 (PDK1), which inhibits the transformation of pyruvate into acetyl-CoA by pyruvate dehydrogenase (PDH) [48]. Consequently, pyruvate is preferentially converted into lactate by LDHA. Lactate is secreted in the extracellular environment, favouring its acidification, which is a phenomenon associated with cancer invasiveness, aggressiveness, angiogenesis, suppression of the immune response, and resistance to therapies [49,50]. Importantly, the concomitantly decreased mitochondrial functioning favours the establishment of an alkaline cytosolic pH, a condition enhancing PFK1 activity [8,51]. Moreover, the downregulation of oxidative metabolism limits the production of reactive oxygen species (ROS) in an adequate range with active proliferation [52], while the limited synthesis of ATP and citrate avoids the negative allosteric inhibition exercised by these molecules on PFK1 and PFK2 [8]. 

Lastly, the Warburg effect in cancer cells is further promoted by mutations in key oncogenes, such as *MYC*, Kristen rat sarcoma viral oncogene homolog (*RAS*), and its downstream targets phosphatidylinositol-3-kinase/Akt/ mammalian target of rapamycin (*PI3K/AKT/mTOR*) signaling pathway, or oncosuppressor genes, such as *TP53* inhibiting glycolysis and Phosphatase and TENsin homolog (*PTEN*) inhibiting PI3K/AKT/mTOR. These proteins are also key regulators of cellular metabolism and energy production, and their mutations may further contribute to the Warburg effect [53,54]. *MYC* is also a key inducer of glutaminolysis [55]. Of note, CS knockdown promotes aerobic glycolysis in cancer cells with the induction of epithelial-to-mesenchymal transition (EMT), a phenotype which was completely reversed by p53 reactivation [56]. Although the high dependence of these cancer cell types on glycolysis correlates with a higher resistance to treatments and a poor survival, as observed in non-small-cell lung cancer (NSCLC) [57,58], it may constitute a vulnerability that could be targeted for therapy. Importantly, resistance to targeted therapies, such as Tyrosine Kinase inhibitors (TKIs), are sustained by aerobic glycolysis and its key oncogenic drivers, such as Ras and its downstream effectors MAPK/ERK and PI3K/Akt [59,60]. 

### 2.2. A Low Citrate Level Is a Driving Force for the Warburg Effect

Citrate is a well-known inhibitor of PFK2 [61]—the key activator of PFK1—as showed, in particular, in ascites cancer cells [3]. PFK2 converts fructose-6-phosphate (F-6-P) into fructose-2,6-bisphosphate (F-2,6-BP), which is a potent allosteric activator of PFK1, and thus of glycolysis [8,62]. PKF2 is a bidirectional enzyme (also named phosphofructo-2-kinase/fructose-2,6-bisphosphatase (PFKFB)) with several isoforms [61,63]. The PFKFB3 isoform, frequently upregulated in cancer cells, has a high kinase activity [62], and thus, it promotes PFK1 and the generation of F-1,6-BP. This molecule is then channelled into the second part of glycolysis, ultimately leading to the production of ATP and pyruvate. Moreover, F-1,6-BP enhances the glycolytic ATP production since it is a well-known allosteric activator of PK [8,64], including the embryonic PKM2 isoform, frequently re-expressed in cancer cells [65,66]. 

Importantly, F-1,6-BP induces an additional feedback loop which may enhance the Warburg effect. Indeed, F-1,6-BP can bind to Son-of-Sevenless-homolog-1 (SoS1) [67], which catalyses Ras activation [68]. In turn, Ras promotes the central mitogen-activated protein kinase (MAPK) pathway (its usual downstream targets), and also PI3K/Akt [69]. Of note, in triple-negative breast cancer (TNBC) cells, it was shown that epidermal growth factor (EGF) signaling activates the first step of glycolysis, and F-1,6-BP directly binds to epidermal growth factor receptor (EGFR), enhancing its activity [70]. Therefore, through EGFR and/or direct binding to SoS1, F-1,6-BP can directly promote Ras/PI3K/Akt signaling, which in turn activates both PFK2/PFKFB3 and HIF-1α, further supporting glycolysis [62,71]. In parallel, F-2,6-BP promotes a truncated gluconeogenesis by inhibiting FBPase [4,8,72].

One of the main characteristics of the Warburg effect is also the concomitant downregulation of the oxidative metabolism, a process that reduces ATP production and favors the export of citrate in the cytosol. This effect is achieved in cancer cells by a cooperation between PI3K/Akt and F-1,6-BP. Indeed, Akt, which promotes LDHA activity [47], concomitantly activates PDK1, a kinase inhibiting pyruvate conversion into acetyl-CoA [40,73]. In parallel, F-1,6-BP reinforces this mitochondrial downregulation and promotes glycolysis by directly inhibiting the activity of the complexes III and IV of the ETC [40]. Consequently, the mitochondrial production of ATP and citrate are limited, and this prevents the inhibition of PFK2/PFKFB3 and PFK1 by high levels of these molecules, especially citrate, a potent inhibitor of PFK2 [3]. Of note, in normal cells, the mechanism of citrate inhibition on PFK2 is non-competitive for ATP [61].

In sum, a vicious cycle is created in glycolytic cancer cells, avoiding high levels of citrate counteracting the major oncogenic drivers of the Warburg effect (namely Akt and HIF-1α) [74]. This regulation results in an enhancement of the activity of the enzymes PFK2, PFK1, LDHA, and PDK1 in a positive feedback loop. Coherently with this, in vitro studies have shown that citrate administered at high concentrations inhibits PFK1 [75,76], PFK2 [3], and also PTEN, the key inhibitor of PI3K/Akt [77,78].

At the same time, low citrate levels downregulate FBPase, favoring a truncated gluconeogenesis, and thus the possible generation of ribose-5-phosphate (R5P) required for nucleotide synthesis [4,72]. If not, high citrate levels would enhance FBPase activity, thus allowing the functioning of the end part of gluconeogenesis, with concomitant downregulation of PFK2 and PFK1 [4,22]. Of note, FBPase is now considered as a onco-suppressor that could inhibits HIF-1α [72]. Interestingly, the low citrate levels observed in malignant mesothelioma cells can be restored by microRNA-126 overexpression, a treatment which efficiently suppresses tumor growth, and inactivates ACLY and the insulin receptor substrate-1 (IRS-1)/Akt pathway [29]. This IRS-1 pathway plays a key role in transmitting signals from the insulin and insulin-like growth factor-1 (IGF-1) receptors to the intracellular pathways PI3K/Akt and MAPK/ERK [79]. 

Remarkably, cancer cells frequently upregulate ACLY, thus accelerating the transformation of citrate to sustain biosynthesis [80,81]. ACLY is an enzyme that is directly activated by Akt [82,83], and by phosphorylated sugars (such as F-6-P, F-2,6-BP and F-1,6-BP) [84]. By upregulating ACLY both via phosphorylation and transcriptional upregulation [85], the PI3K/Akt pathway favors histone acetylation [86], leading to increased lipid and cholesterol synthesis. Consequently, the engagement of glycolysis (sustained by glucose abundance) is coupled with the rapid turnover of molecules such as citrate, which sustains biosynthesis, a turnover favoured by ACLY upregulation and downregulation of OXPHOS. As a result, the glycolytic pathway cannot be inhibited by ATP and citrate, while the biosynthesis sustained by the Warburg effect is not arrested. In addition, ACLY can promote the EMT phenotype in cancer cells [87] (presumably by regulating Snail expression) and stemness [80,81,88]. In this setting, PI3K/Akt and ACLY activate the canonical WNT/β-catenin pathway, which promotes EMT and the development of fibrotic tumor stroma, as well as exhaustion of immune response and cell cycle progression [89,90,91]. Of note, EMT has also been related to the emergence of cancer stem cells (CSCs) [92], which could, therefore, be supported by the PI3K/Akt/ACLY axis. Accordingly, ACLY knockdown reverses EMT and cancer stemness, especially by reducing Snail expression [87,88]. In line with this, citrate inhibits CSCs survival, and it reverses EMT in an in vivo lung cancer model, especially in conjunction with chemotherapy, as assessed by an increase in E-cadherin expression and a reduced expression of Vimentin and Snail [88]. In addition, it has been shown that citrate is able to induce cell death of cells showing the CSC phenotype CD44 high/CD24 low [77].

From a larger perspective, the metabolism of cancer cells is coupled with the cell cycle progression since some key metabolic enzymes are multifunctional enzymes, also acting as regulators of the cell cycle [93]. Particularly, PFKFB3, PKM2, and GAPDH periodically translocate to the nucleus during the cell cycle in order to regulate the expression of oncogenes or cell cycle regulators [38]. F-2,6-BP promotes G1/S phase transition, as it induces the degradation of the suppressor p27 [62]. In addition, GAPDH, which induces resistance to apoptosis, likely by promoting the overexpression of the key anti-apoptotic factor B-cell lymphoma-extra large (Bcl-xL) protein [94], accelerates cell cycle progression towards mitosis [95].

## 3. The Central Place of Citrate in Cancer Cells Relying on Oxidative Metabolism

### 3.1. Cancer Cells Are Not Inherently Glycolytic

In vitro studies have shown that numerous cancer cells do not rely mainly on the Warburg effect but predominantly on an oxidative or intermediate metabolism [30,96,97]. This has been especially observed in cells growing in lipid-rich environments (such as triple negative breast cancer cells or ovarian cancer cells developing in the peritoneum), as well as in cancer stem cells (CSCs) [31,98]. These cells produce energy mainly through FAO [97,99], whose induction may be dependent on the activation of proliferator-activated receptor gamma coactivator 1-alpha (PGC1-α) by acetylation [100,101] and of adenosine monophosphate activated protein kinase (AMPK) [102]. Moreover, in aggressive cancer cells, it is not infrequent to observe the upregulation of mitochondrial enzymes, such as CS [103,104], or mitochondrial transporters, such as some translocases of the inner and outer mitochondrial membrane (TIMM and TOMM) [105]. Interestingly, the mitochondrial enzyme phosphoenolpyruvate carboxykinase (PCK2), which sustains gluconeogenesis, has been found to be more frequently upregulated in lung adenocarcinomas than in epidermoid tumors [58], and PCK2 expression is associated with a better prognosis compared to the expression of glycolytic expression of GLUT1 [58]. Of note, a metabolic cooperation can be established between hypoxic and non-hypoxic cancer cells in tumors, thus helping to spare glucose for the most glycolytic cells [106]. To achieve this regulation, lactate released by glycolytic cancer cells expressing MCT4 can be recycled by oxidative cells expressing MCT1. Here, lactate is converted into pyruvate by a reverse LDH-A reaction, and in turn pyruvate fuels the TCA cycle to produce energy and sustain biosynthesis [107,108]. In this setting, cancer cells chronically exposed to extracellular acidosis (due, for example, to excess protons secreted by glycolytic cancer cells that saturate bicarbonate buffer) would reprogram their metabolism, arresting glycolysis (proton H^+^ is a well-known inhibitor of PFK1 [8,51]), and enhancing reductive carboxylation of glutamine sustaining FAS and promoting FAO [109]. The latter pathway provides acetyl-CoA for the functioning of the TCA cycle and for the acetylation of mitochondrial proteins, particularly of complex I, this process restraining ATP and ROS production [109]. Of note, citrate, OAA and malonate are well known inhibitors of succinate dehydrogenase (SDH) (or complex II), particularly in liver, brain, cardiac and muscle cells [110,111,112]. However, the modulation of SDH by these molecules in cancer cells remains to be studied. 

Prostate cancer (PCa) (presumably the epithelial cancer with the most favourable prognosis), exhibits an oxidative metabolism [113], but may switch to the Warburg effect at the advanced metastatic stage [113]. FAO has been reported to be the dominant energetic pathway in PCa, which show a very low affinity for 2-deoxy-2-fluoro-D-glucose (FDG) (an indicator of glycolytic flux) in a positron emission tomography (PET) scan [114]. PCa cells promote ACO2 activity, favoring the oxidation of citrate in the TCA to produce energy (Figure 1). ACO2 upregulation has been identified as a key event in prostate carcinogenesis [113], a process promoted by a zinc deficiency due to the downregulation of the zinc transporter [115]. Accordingly, while the accumulation of zinc in benign prostate cells inhibits citrate oxidation and favours citrate accumulation in prostate tissue [113], lower levels of citrate, zinc, aspartate and spermine have been observed in PCa in comparison to non-cancer epithelium [116,117].

### 3.2. Citrate Constitutes a Driving Force also in Cells Relying on Oxidative Metabolism

As we have seen, some cancer cells can engage glutamine oxidation and/or FAO to sustain energy production and their growth, particularly in a microenvironment lacking glucose and/or rich in lipids [97,101], and/or submitted to chronically acidic pH conditions [109]. In this context, pyruvate carboxylase (PC), which is frequently upregulated, redirects the carbon flux (coming from glutaminolysis and/or FAO) towards gluconeogenesis providing the supply of precursors molecules for nucleotide synthesis [22,72]. Gluconeogenesis is the reverse pathway of glycolysis, which is physiologically activated (as showed in normal hepatocytes) by four enzymes: PC, PCK, fructose-1,6-bisphosphatase (FBPase), and glucose-6-phosphatase (G6Pase). 

FAO sustains a high production of acetyl-CoA and/or of KBs (including acetone, which can feed gluconeogenesis). Acetyl-CoA (derived from FAO or KB degradation) is conjugated to OAA (produced by PC from pyruvate) to form citrate. The low affinity of ACO2 for citrate regulates the carbon flux in the TCA cycle, thus adapting ATP production to the cell requirements and avoiding its overproduction. The citrate, in excess, is exported out of mitochondria into the cytosol [42,43,44,45]. Here, citrate is converted back by ACLY into acetyl-CoA and OAA (Figure 1). The latter can sustain gluconeogenesis by conversion into phosphoenolpyruvate (PEP), a reaction catalysed by PCK, which has both a cytosolic isoform (PCK1) and a mitochondrial isoform (PCK2). While cytosolic PCK1 mainly converts citrate-derived OAA into PEP, mitochondrial PCK2 converts pyruvate-derived OAA (produced by PC) into PEP, which is further exported into the cytosol by a specific transporter [118]. Of note, mitochondrial OAA can be furnished by PC, but also by the recycling of cytosolic OAA (derived from citrate) converted back by MDH into malate, a molecule that can enter mitochondria.

Similarly to what is observed in normal cells, it is possible that the high cytosolic level of citrate—derived from FAO—promotes, in some cancer cells, the inactivation of glycolysis while concomitant gluconeogenesis is activated [7]. However, the upregulation of ACLY may prevent or attenuate this so-celled Randle cycle regulation, by inducing a continuous leak of citrate, which favours a truncated gluconeogenesis because FBPase is inactivated. Therefore, some of gluconeogenesis intermediates can be redirected towards the serine–methionine pathway and the non-oxidative PPP to sustain biosynthesis. As a result, a low citrate level would concomitantly promote futile cycles, gluconeogenesis and glycolysis, FAO and *de novo* FAS. Further studies are required to precisely establish the metabolism and regulation of oxidative cancer cells in various environmental conditions.

## 4. The Possible Role of Citrate in the Metabolism of the Microenvironment

There is a variety of non-cancerous cells in the TME, such as cancer-associated fibroblasts (CAFs), endothelial cells, macrophages, and immune cells. The latter have numerous subgroups, and schematically includes cells fighting cancer cells, such as cytotoxic CD8^+^ T lymphocytes and pro-inflammatory M1 macrophages, and also cells promoting cancer development (i.e., pro-tumoral immune cells), such as myeloid-derived suppressor cells (MDSCs), regulatory T cells (Tregs), and M2 anti-inflammatory macrophages (for a review, see [119]). Cancer cells deprive TME of nutrients, particularly glucose and amino acids. At the same time, cancer cells can secrete different waste products (lactate, nitric oxide (NO), polyamines, adenosine, and H^+^)**,** which can drive exhaustion (non-responsiveness) of M1 macrophages and cytotoxic T cells. Concomitantly, immunosuppressive cells, which preferentially rely on FAO, are activated [120,121,122,123,124]. Of note, hypoxia and HIF-1α promote the formation of a fibrotic tumor stroma [125]. Lactate secreted by glycolytic CAFs (a process named “the reverse Warburg effect”) can sustain the metabolism of oxidative cancer cells [126]. Thus, strategies targeting the recycling of waste products or redirecting nutrients towards cytotoxic immune cells appear as rational interventions to counteract the growth of cancer tumors [127]. Remarkably, peritoneal tumor xenografts in mice (B16 melanoma or ID8 ovarian carcinoma) promote glycolysis and FAO in peritoneal resident macrophages with a high mitochondrial production of itaconate [128]. Itaconate is a potent anti-inflammatory molecule derived from *cis*-aconitate, produced by the aconitate decarboxylase (ACOD) encoded by the immune-responsive gene-1 (*Irg1*) [129]. In tumors, the mechanisms by which aconitate production is enhanced and by which it promotes tumor growth are still not clear and warrant further investigation.

The citrate available in the TME appears to be essential to support cancer cell growth. As we have seen, in cancer cells, citrate is generated by the activity of the CS and/or the carboxylation of AKG (especially in cells with dysfunctional OXPHOS or TCA cycle) [39]. However, several experiments have made it possible to estimate that up to one third of the total intracellular citrate pool in cancer cells may also be derived from the direct uptake of extracellular citrate, a process promoted further by glucose deprivation [130]. Experimental studies showed that physiological concentration of citrate (200 µM) can sustain the proliferation of various cancer cells, such as prostate, pancreatic and gastric ones [130,131]. Interestingly, citrate concentrations of up to 5 mM can stimulate lipid synthesis and histone acetylation in HepG2 cells, but higher concentrations (10 mM or more) decreased both acetylation and ACLY expression [132]. Factors influencing the blood concentration of citrate (which is normally around 100–150 µM [133]) have not been well characterized up to now. In theory, it likely depends on the daily intake, the secretion of citrate by body reserves (approximately 90% of citrate is located in mineralized tissues), and the citrate consumption in various biological processes, particularly bone formation [134]. 

## 5. The Anti-Cancer Effects of Citrate at High Dosages in Preclinical Experiments

### 5.1. In Vitro Studies

In various cell lines, a high concentration of citrate—generally above 10 mM—inhibits the proliferation of cancer cells in a dose dependent manner. As showed in Table 1, this anti-cancer effect involves various mechanisms such as the inactivation of PFK1, the inhibition of glycolysis and ATP production, and the stimulation of apoptotic cell death by various processes (in particular, the activation of pro-apoptotic proteins and various caspases (2, 3, 8, and 9), and the decreased expression of the two key anti-apoptotic factors, Bcl-xL and Mcl-1 [75,76]). Of note, the levels of glucose-derived citrate are decreased by approximately 25% in Bcl-xL-expressing cells compared to control ones [135], and the addition of citrate to these cells leads to increased protein N-α-acetylation and sensitization to apoptosis [135]. Importantly, citrate at high concentration (10 mM) also inhibits the insulin-like growth factor-1 receptor (IGF-1R) pathway, which promotes the downstream activation of PI3K/Akt/mTOR and MAPK pathways [77]. In addition, a high dose of citrate activates PTEN, the key phosphatase inhibiting the PI3K/Akt pathway [77,78]. Furthermore, citrate increases the sensibility of cells to chemotherapy (in particular, cisplatin) and to Bcl-xL inhibitors (ABT 737 or siXL1), also increasing the levels of interleukin-1β, interleukin-8 and tumor necrosis factor (TNF).

### 5.2. In Vivo Studies

Citrate inhibits the growth of several xenograft cancer models in mice, increasing the response to chemotherapy. Indeed, daily intra-peritoneal (i.p.) injection of sodium citrate for 4 weeks (15 to 30 mg/kg/day) reduced tumor development in a gastric cancer model (SGC-7901 cells in nude mice), partly by promoting tumor apoptosis [75]. Similarly, in murine tumor models of human osteosarcoma and fibrosarcoma, i.p. injections (two times per week) of citrate (50 to 100 mg/kg), caffeine (50 to 100 mg/kg), and caffeine citrate (100 to 200 mg/kg) reduced tumor growth (with caffeine citrate showing the stronger effect), and all molecules potentiated the anti-tumoral effect of cisplatin treatment [142]. Oral citrate administration also impacts tumor growth. Indeed, oral gavage of citrate sodium (4 g/kg twice a day) for several weeks (4 to 7 weeks) significantly regressed tumors in various murine models, such as subcutaneously implanted syngeneic pancreatic tumor (Pan02), human lung adenocarcinoma (A549 cells) xenografts in nude mice, Ras-driven lung cancer in genetically engineered mouse (GEM), and breast cancer driven by human epidermal growth factor receptor 2/(Her2/Neu) in GEM [77]. Regression of tumors was frequently associated with differentiation and abundant leukocyte infiltration, predominantly constituted of T lymphocytes. Interestingly, plasma citrate levels of these chronically citrate-treated mice were approximately 3 mM, roughly eight times higher than the ones recorded for non-citrate treated mice [77]. A recent study showed that citrate also suppresses growth of PCa xenograft tumors in mice [145]. Of note, in a pancreatic cancer-xenograft murine model, 14 daily doses (500 mg/kg/day) of oral citrate induced the neutralization of TME acidity and potentiated the therapeutic effect of an oral administration of active 5-fluoro-uracil derivative [146]. 

## 6. Discussion and Therapeutic Perspectives

In sum, the central role of citrate in the metabolism of cancer cells, which we first outlined some years ago [1], has been confirmed by various studies [147,148]. Indeed, citrate provides acetyl-CoA (supporting various processes such as protein acetylation regulating enzyme activities, histone acetylation regulating gene expression, and lipid synthesis required for membrane formation) and also OAA (which can sustain biosynthesis or supply energy through TCA cycle coupled with OXPHOS). In addition, contrary to what was observed in normal cells, in cancer cells, the upregulation of ACLY concurs to maintain a low cytosolic level of citrate, favoring further the enhancement of glycolysis (which would be inhibited by high citrate) and the activation of oncogenic drivers, such as the PI3K/Akt and WNT/β-catenin pathway, which promotes aggressiveness, EMT and de-differentiation [77,87,91,144,145,149]. As we have seen, we proposed that the impediment of the negative feedback exercised by citrate on PFK1 and PFK2 is an essential factor of the Warburg effect functioning, allowing high production of F-1,6-BP. This high production promotes Ras/PI3K/Akt signaling [67,68,69], which in turn activates both PFK2/PFKFB3 [62,150] and HIF-1α [151], further supporting glycolysis. Moreover, PI3K/Akt downregulates the TCA cycle coupled with OXPHOS functioning [73], and F-1,6-BP directly inhibits OXPHOS [40]. At the same time, ACLY activity is enhanced by Akt [82,83] and phosphorylated sugars [84]. 

Although the specific effects of citrate on all the different cell types populating the TME remains to be studied, we suggest that immunosuppressive cells, which preferentially rely on FAO [120,123,124], could release citrate at physiologic concentrations in the TME, thus sustaining cancer cell growth. Of note, these immunosuppressive cells may recycle the lactate secreted by tumor cells or CAFs [126]. This could create a metabolic cooperation between immunosuppressive cells and cancer cells, since lactate excreted by cancer cells would sustain the secretion of citrate by immunosuppressive cells, these cells also secreting itaconate promoting cancer growth. Further in vitro studies could help to clarify the impact of these putative cycles on cancer growth. The development of new biological methods and imaging techniques, allowing the measurement of molecule concentrations in tumors, would help in analysing these possible metabolic adaptations in the TME. 

From a therapeutic perspective, two strategies have been proposed to target citrate metabolism. In the first, citrate uptake by cancer cells could be blocked by inhibiting the membrane transporter *pmCiC* (a specific transporter mediating citrate uptake by cancer cells), specifically by using gluconate. This strategy efficiently counteracted tumor growth in a human pancreatic xenograft murine model [130]. In the second (that we proposed), cancer cells and TME could be flooded by high concentration of citrate (30 to 50 times the physiological level), with the objective of inhibiting glycolysis in glycolytic cancer cells by various mechanisms that have previously been described, likely based on the increase in cytosolic citrate level promoting PFK and glycolysis inhibition. As we have stated, dichloroacetate, which inhibits PDK1 and restores drug sensitivity in paclitaxel-resistant lung cancer cells (A549), increases cytosolic citrate [28], while ACLY inhibition, which reduces tumor growth in various models, likely increases citrate level [87]. Furthermore, sodium citrate is also a basic salt, which similarly to bicarbonate, may buffer acidity in TME. This effect favours the penetration of chemotherapy drugs (such as doxorubicin) in cancer cells, also improving the efficacy of mTORC1 inhibitors (rapamycin), and the response to immunotherapies [152,153,154]. The buffering of extracellular acidity could also counteract cancer cells relying on oxidative metabolism, in particular FAO, which is promoted in cancer cells by chronic acidity [109].

As Mycielska and Geissler commented [155] ‘both the “low” and “high” citrate uptake approaches do make sense’. We agree that strategies altering the intracellular citrate levels in either direction should be further explored. Short cycles of “metabolic interventions” targeting citrate may appear preferable compared to prolonged interventions, since the metabolism of various cancer cells is plastic, and some clone cells could adapt drugs or metabolic inhibitory strategies, shifting, for example, from a glycolytic to an oxidative metabolism (and vice versa) or bypassing the inhibition of a chronically administered strategy. In this setting, an in vitro study reported the occurrence of a resistant clone of metastatic PCa cancer cells (PC3) under prolonged 10 mM citrate exposure (more than two weeks). This process was associated with a shorter PKF1 form (insensible to citrate regulation) and with the upregulation of autophagy [156]. Of note, this survival process—recycling superfluous molecules (proteins and lipids)—requires functional mitochondria for ATP production derived from FAO [157]. However, the effects of citrate on PCa are controversial, taking into account that a recent study reported that citrate activates autophagic cell death via downregulation of the Akt/mTOR pathway, also suppressing growth of PCa xenograft tumors in mice [145]. Additionally, a prolonged inhibition of *pmCiC* could be bypassed by increasing the uptake of acetate, which is another source of acetyl-CoA [158], or by producing acetyl-CoA directly from glycolysis through transketolase-like 1 activity [159]. While waiting for new methods assessing the metabolism of a specific tumor in vivo and its vulnerabilities for personalized specific inhibitions, we think that the *citrate strategy* we proposed should be tested during brief periods to increase the sensibility or response to cytotoxic treatments including new anti-cancer therapies.

For clinical tests, it should be mentioned that citrate has a very low toxicity (see “citrate” PubChem CID 311, at https://pubchem.ncbi.nlm.nih.gov/, accessed on 17 June 2021), as confirmed also by in vivo studies [145], because it is an endogenous metabolite with a complete and rapid metabolism, and thus a very short half-life [160]. However, if administrated in excess, citrate could cause hypocalcaemia, muscle spasms, convulsions, and also a risk of haemorrhage due to its chelating properties of calcium and other divalent cations. These effects can be treated urgently and at best prevented by administration of calcium chloride. By extrapolating the results of a preclinical model [77], the active dose in man would be likely much lower than the one inducing the adverse effects. Clinical trials should determine the mode and duration of citrate sodium administration, its toxicity, and its efficiency. Knowing that numerous patients worldwide have incurable cancers supported by aerobic glycolysis and key oncogenic drivers (such as IGF-1R, Ras/PI3K/Akt, HER2/neu, WNT/β-catenin, TME acidity and EMT) [145,161,162], all pathways efficiently counteracted by citrate sodium in preclinical studies, we strongly believe that the *citrate strategy* we have proposed since many years [25] should now be considered for clinical trials. In particular, this strategy could increase both the sensitivity to standard chemotherapy drugs and to targeted therapies, whose resistance is mainly supported by the Warburg effect and its oncogenic drivers.

## Figures and Tables

**Table 1:** Literature review of the main studies describing the effects of citrate (≥10 mM) on cancer cells.

Cells	Cancer Type	In Vivo/In Vitro	Citrate Targets/Citrate Effects on Cells	Year	Ref.
MSTO-211H cells	Mesothelioma cells	In vitro	Decreased expression of anti-apoptotic protein Mcl-1 and Bcl-xL. Depletion of ATP. Increase in the cisplatin antitumor effect.	2009	[25]
B16 cells	Murine melanoma	In vitro	Combined treatment with UVB increases c-Jun and p38 phosphorylation and the activity of caspase-3 and -9.	2009	[136]
HeLa cells	Adenocarcinoma	In vitro	Increase in the protein N-alpha-acetylation. Sensitivity to apoptotic stimuli.	2011	[135]
BGC-823 cells SGC-7901 cells	Gastric cancer	In vitro	Decreased expression of anti-apoptotic protein Mcl-1.	2011	[26]
Tet21N cells SKNAS cells SKNSH cells SK-N-BE(2) cells U1810 cells	Neuroblastoma Neuroblastoma Neuroblastoma Neuroblastoma Lung cancer	In vitro	Activation of the apical caspases-8 and -2. Increased release of the cytochrome C.	2012	[137]
C6 cells	Glioblastoma	In vitro	Inhibition of the number of vascular branching points. Inhibition of the length of vascular tubules. Inhibition of angiogenesis.	2012	[138]
SKOV3 cells IGROV1-R10 cells	Ovarian cancer	In vitro	Decreased expression of the anti-apoptotic protein Mcl-1. Increase in the efficacy of ABT 737 treatment.	2013	[27]
U937 cells	Acute Monocytic Leukemia	In vitro	Increase in caspase-3 and -9 activities. Decreased expression of anti-apoptotic protein Bcl-2.	2013	[139]
MGC-803 cells	Gastric cancer	In vitro	Increased expression of the pro-apoptotic protein Bax. Inhibition of the PFK activity. Decrease in lactate and ATP production.	2016	[76]
SGC-7901 cells	Gastric cancer	In vitro and in vivo	Inhibition of the PFK1 activity. Decreased tumor growth and increased apoptosis (increased cytC release and Bax expression, reduced Bcl-2).	2016	[75]
A549 cells Pan02 cells Her2/Neu model	Lung cancer Pancreatic cancer Breast cancer model	In vitro and in vivo	Inhibition of IGF-1R/AKT pathway via PTEN. Inhibition of tumor growth in Ras-driven lung cancer, Pan02 xenograft and Her2/Neu models.	2017	[77]
EC109 cells	Oesophageal cancer	In vitro	Increase in apoptosis.	2017	[140]
AGS cells	Gastric cancer	In vitro	Increase in the levels of interleukin-1β, IL-8 and TNF. Increased activity of caspase-3 and -9.	2018	[141]
HOS & LM8 cells HT1080 cells	Osteosarcoma Fibrosarcoma	In vitro and in vivo	Enhancement of cisplatin anti-tumor effect.	2019	[142]
PSC cells	Pharyngeal carcinoma	In vitro	Cell cycle arrest at the G2/M phase. Stabilization of cyclinB1-CDK1 through p85α-PTEN.	2019	[78]
MCF-7 cells	Breast cancer	In vitro	Increase in the efficacy of radiation therapy.	2020	[143]
HMV-II cells	Melanoma	In vitro	Inhibition of cancer cell proliferation. Reduction in β-catenin levels.	2020	[144]
HepG2 cells	Hepatoma	In vitro	Inhibition of the ACLY-mediated H4 acetylation and lipid deposition.	2020	[132]
PCa cells	Prostate cancer	In vitro and in vivo	Induction of cell death through autophagy modulation. Inhibition of tumor growth in a xenograft model.	2021	[145]
Panc-1 cells	Pancreatic cancer	In vivo	Neutralization of TME acidity. Potentiation of anti-tumor effect of 5-FU derivative. Reduction in tumor growth (xenograft model).	2021	[146]
